# Contributing Factors to Medication Administration Errors Among Novice Registered Nurses: An Integrative Review

**DOI:** 10.1111/jocn.17721

**Published:** 2025-03-03

**Authors:** Chelsea Webb, Elissa Dabkowski, Karen Missen, Amanda Missen

**Affiliations:** ^1^ Institute of Health and Wellbeing, Federation University Australia Churchill Victoria Australia; ^2^ Latrobe Regional Health Traralgon Victoria Australia

**Keywords:** error, graduate, medication, medication administration, novice, nurse, patient safety, registered nurse, safety

## Abstract

**Aim:**

The aim of this review was to explore the influencing factors that contribute to medication administration errors (MAEs) made by novice registered nurses (NRNs).

**Background:**

MAEs are a significant yet preventable risk to patient safety in healthcare settings, compromising both patient health and care quality. Evidence suggests that NRNs are more prone to MAEs, highlighting the importance of exploring the contributing factors to develop effective prevention strategies.

**Design:**

An integrative review.

**Methods:**

An electronic literature search was conducted in which articles were restricted to peer‐reviewed, full‐text articles published in the English language between January 2013 and December 2023. Quality assessments and data syntheses were conducted by two independent authors.

**Data Sources:**

CINAHL Complete, MEDLINE, APA PsycArticles, APA PsycInfo, PubMed, Cochrane Library and Web of Science.

**Results:**

Eleven studies met the inclusion criteria. The main influencing factors identified in this review were intrinsic factors (lack of confidence, reduced coping skills and reluctance to seek assistance) and extrinsic factors (time pressures, hectic workloads, low staffing and high‐stress environments). Clinical, educational and research implications were also identified.

**Conclusion:**

This integrative review explored the various influencing factors contributing to MAEs by NRNs in healthcare settings. These included gaps in pharmacological knowledge, limited simulation‐based learning and challenges in using electronic medical records. Addressing these issues through targeted education and increased simulation experiences in undergraduate curricula could enhance NRNs' competence and confidence.

**Reporting Method:**

Preferred Items for Systematic Reviews and Meta‐Analysis (PRISMA) guidelines.

**Patient or Public Contribution:**

No patient or public contribution was made due to the study design.

**Implications for the Profession and/or Patient Care:**

Understanding the factors behind medication administration errors among new nurses helps organisations develop strategies to address these issues, reducing patient harm and enhancing nursing practice. Our findings offer recommendations to improve nursing education worldwide.

**Summary**

- Medication administration errors (MAEs) pose a significant risk to patient safety, leading to potential harm and adverse outcomes.
- Novice registered nurses are particularly susceptible to MAEs
- Addressing intrinsic and extrinsic factors through targeted education and simulation‐based training may help to reduce the incidence of MAEs amongst novice registered nurses.

## 1 Introduction

Medication administration errors (MAEs) pose a significant threat to patient safety, encompassing various incidents related to the prescription, dispensing and administration of medications (Fernandez et al. 2023). These MAEs can result in adverse drug events, prolonged hospital stays, and in severe cases, fatalities (Fernandez et al. 2023; World Health Organization 2017). The role of nurses in medication management is crucial, as they are responsible for administering medications, monitoring their effects and reporting any issues (Alomari et al. 2018). Research indicates a direct correlation between nurses' pharmacological knowledge and their length of experience, highlighting that those with less than 1 year of work experience are more prone to making MAEs compared to their more experienced counterparts (Kerari and Innab 2021; Schroers et al. 2021). Therefore, it is imperative to comprehend the factors contributing to MAEs made by novice registered nurses in order to develop effective prevention strategies and enhance patient safety in the clinical setting.

## 2 Background

The National Coordinating Council for Medication Error Reporting and Prevention [NCCMERP] (2023) in the United States of America defines a medication error as any preventable event that may contribute to inappropriate medication use or cause harm to a patient while the medication is under the control of a healthcare professional, patient, or consumer. Addressing MAEs has long been a focal point in health care, posing a significant global challenge with an estimated annual cost of $42 billion USD, as reported by the World Health Organization (WHO) in 2017 (World Health Organization 2017). Notably, nurses experience a higher rate of MAEs compared to other healthcare professionals, and research indicates that these errors often have multifaceted and interconnected origins (Alomari et al. 2018; Fernandez et al. 2023; Mostafa et al. 2020).

In a 2021 integrative review conducted by Kerari and Innab, it was found that the occurrence of MAEs in healthcare settings is closely linked to factors such as nurses' educational background, years of experience and involvement in training programmes. The authors of this review suggest that novice registered nurses are more prone to making MAEs compared to their more experienced counterparts (Kerari and Innab 2021). This raises concerns about the readiness of novice registered nurses for practical application in clinical settings and their ability to translate theoretical knowledge into practice (AlMekkawi and El Khalil 2020; Missen et al. 2016). Despite rigorous academic nursing programmes, the real‐world dynamics of healthcare environments can be overwhelming for novice registered nurses, resulting in elevated stress levels and diminished cognitive abilities (AlMekkawi and El Khalil 2020; Missen et al. 2015). Additionally, the fast‐paced nature of healthcare settings and the need for multitasking can lead to distractions and fatigue, affecting novice registered nurses' concentration and attention to detail (Treiber and Jones 2018a).

While numerous studies have explored the readiness of novice registered nurses for practice, few have delved into the factors influencing MAEs in this particular cohort. The basis of this review is to explore existing literature on medication errors made by novice registered nurses with less than 2 years of work experience in healthcare settings. This review aligns with the WHO's strategic framework of the *Global Patient Safety Challenge*, which identifies the education and training of new healthcare workers as an essential element of medication safety (World Health Organization 2023).

## 3 Research Aim

The overarching aim of this review was to explore the factors influencing medication administration errors made by novice registered nurses. The research question guiding this review is:

1. What are the influencing factors in contemporary literature that contribute to novice registered nurses making medication administration errors?

For the purposes of this review, we will refer to all graduate nurses and variations to novice or newly registered nurses.

## 4 Methods

### 4.1 Design

This integrative review was guided by the methodology outlined by Whittemore and Knafl (2005). An integrative review was the preferred methodology to address the research question due to the incorporation of a diverse range of research designs. This methodology incorporates a five‐step process which includes problem identification, literature search, data evaluation, data synthesis and presentation (Whittemore and Knafl 2005). The problem identification was discussed in the background section. This review was reported using the Preferred Items for Systematic Reviews and Meta‐Analysis (PRISMA) guidelines (Page et al. 2021).

### 4.2 Literature Search

The authors consulted a research librarian regarding the search strategy before commencing the review. A Boolean search strategy was used, which included MeSH headings, key concepts and variations and truncated symbols of the terms, “nurs*”, “graduat*” “medication” and “error*”. The formal literature search was conducted between December 2023 and January 2024 of four EBSCO databases (CINAHL Complete, MEDLINE, APA PsycArticles and APA PsycInfo). Further databases searched included PubMed, the Cochrane Library and Web of Science. Additionally, the authors examined Google Scholar, Connected Papers (an online visual tool) and manually searched the reference list of systematic reviews. Limiters were applied to the search to include peer‐reviewed, full‐text articles published in the English language between January 2013 and December 2023. This date range was selected as the authors were only interested in contemporary literature pertaining to medication administration errors, given the rapid advancement of healthcare technology. The search results were uploaded to Endnote and exported to Covidence, a software program to manage systematic reviews (Veritas Health Innovation 2021). The last search of this review was conducted on 17th January 2024.

### 4.3 Screening and Eligibility

After de‐duplication, a title and abstract screen of the search results in Covidence was conducted independently by two authors (C.W. and E.D.). In the event of disagreement, a third author (K.M.) moderated this process to reach consensus. Articles of a qualitative, quantitative or mixed‐methods study design were considered if they addressed the research question. The authors collectively decided to include peer‐reviewed, primary articles and exclude grey literature such as discussion papers, editorials or conference abstracts. Dissertations were excluded from this study. Studies were included if the population of interest was novice registered nurses, defined as having less than 2 years of work in any healthcare setting following the completion of an undergraduate nursing degree. Articles pertaining to undergraduate students and/or registered nurses with more than 2 years of work experience were excluded, unless data related to novice registered nurses were discernible. The approved screened records were obtained in full text by one of the authors (C.W.) and further examined to determine relevance to the research question. Articles were included if data collection pertained to medication errors that were committed by novice registered nurses and/or their influencing factors. The medication errors could have been experienced in either a simulation or a clinical setting.

### 4.4 Data Evaluation

A critical appraisal of research literature was performed independently by two authors (C.W. and E.D.). Included papers of a qualitative design were evaluated using the Critical Appraisal Skills Programme (CASP) qualitative studies checklist (Critical Appraisal Skills Programme 2018). The Mixed Methods Appraisal Tool (MMAT) was used to evaluate studies of a mixed‐methods design (Hong et al. 2018), with the Joanna Briggs Institute (JBI) checklist for quasi‐experimental studies (Tufanaru et al. 2020) and the JBI checklist for analytical cross‐sectional studies (Moola et al. 2020) used as applicable. Although a summative quality appraisal score is not always applicable, the authors opted to include this in the data summary tables for transparency.

### 4.5 Data Synthesis

Data were extracted and synthesised according to guidelines from Whittemore and Knafl (2005). The obtained data from the primary articles included the author, year and country, study aim, study design, population and settings, measures/analysis used in the study and main findings. These headings were iteratively developed by the authors, in which the main findings were categorised based on the research questions and pertained to identifying influencing factors contributing to novice registered nurses making MAEs, the instruments/tools used to identify these factors, and explore the educational and/or research implications. As per Whittemore and Knafl (2005), three separate data display matrices were developed to display the data from each of the qualitative, quantitative and mixed‐methods study designs.

## 5 Results

### 5.1 Search Outcome

**FIGURE 1:**
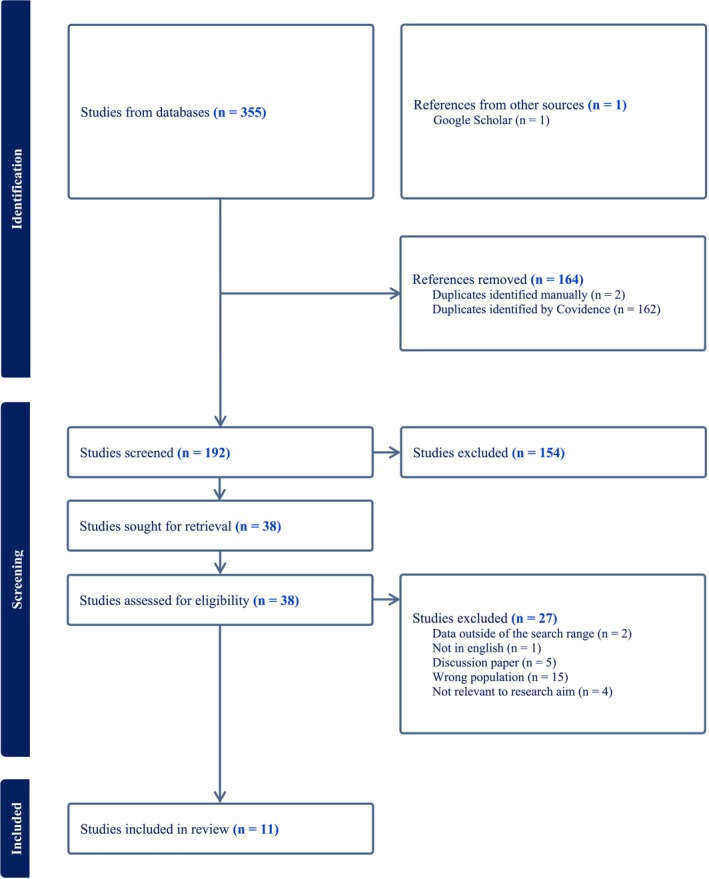
PRISMA flow chart of search strategy (Page et al. 2021). [Colour figure can be viewed at wileyonlinelibrary.com]

From the initial database search, a total of 355 citations were uploaded into Covidence, with an additional article added from Google Scholar. During the de‐duplication process, 164 records were removed, leaving a total of 192 citations. Two researchers assessed the titles and abstracts of the 192 citations against the predetermined eligibility criteria, reducing the selection down to 38 articles for full‐text review. Of the 38 articles, 27 studies did not meet the eligibility criteria. This search process is summarised in Figure 1, resulting in 11 articles that met the inclusion criteria for this review.

### 5.2 Article Characteristics

Four of the studies that met our eligibility criteria were conducted in Australia (Murray et al. 2019; Sahay et al. 2015; Sahay and Willis 2022; Wilson et al. 2016) with a further four studies conducted in the United States of America (Chan and Burns 2021; Myroniak and Elder 2021; Treiber and Jones 2018a, 2018b). One study was carried out in Sweden (Willman et al. 2021) and another in New Zealand (Lim and Honey 2017). Fernandez et al. (2023); Fernandez et al. (2023) had participants from four different countries: Australia, South Africa, India and Turkey. Having six countries represented in this review highlights the global issue of novice registered nurses committing medication administration errors and the impact it has on patient safety worldwide. The methodologies employed across the 11 articles varied, with four mixed‐method studies, three quantitative studies (including two cross‐sectional studies and one quasi‐experimental study), and four qualitative studies. The study sample size varied in the 11 articles from 11 to 576 participating graduate nurses. All novice registered nurses had graduated with a bachelor's degree in nursing and had less than 24 months of clinical experience. Table 1 displays the data matrix of qualitative findings, while Tables 2 and 3 display the data matrices of quantitative and mixed‐method findings.

**TABLE 1:** Data matrix of qualitative findings.

Author/year/country/title	Study aim	Study+design	Population & setting	Measures/analysis	Main findings	Risk of bias tool
Murray et al. (2019) Australia ‘New graduate nurses' understanding and attitudes about patient safety upon transition to practice’	To explore the transition experience of newly graduated nurses with particular attention to patient safety	Qualitative descriptive	11 graduate nurses employed in tertiary hospitals in Western Australia	Individual semi‐structured interviews Thematic analysis	Major theme: ‘Patient safety and insights’, ‘Time management’, ‘Making a mistake’, ‘Experiential learning’ and ‘Transition’. Two subthemes: ‘Apprehension’ and ‘Confidence and doubt’ Graduate nurses reported internal conflict when witnessing errors in medication administration by senior nurses. Graduate nurses struggled to speak up for themselves to request RNs to follow best practices. Participants also discussed different ward cultures impacting patient safety and medication administration, such as senior nurses often stating that their ward did not check at the bedside for APINCH, (**A**nti‐infective, **P**otassium, **I**nsulin, **N**arcotics, **C**hemotherapeutic agents and **H**eparin) medication, despite this being best practice. Participants expressed a deficiency in knowledge relating to types of medications and their interactions. Participants exposed a lack of insight regarding the graduate nurse role in education on medications. Participants reported an underlying fear of making a mistake, that would result in patient deterioration.	CASP qualitative checklist 90%
Sahay and Willis (2022) Australia ‘Graduate nurse views on patient safety: Navigating challenging workplace interactions with senior clinical nurses’	To explore the negative experiences of nurses' interaction with senior nurses and the implications for safe patient care	Qualitative exploratory study	18 graduate nurses	Individual semi structured interviews Thematic analysis	Major theme: ‘Navigating workplace challenges’ Two sub‐themes: ‘Processing unsupportive nurse Behaviour’ and ‘Responding to nurse deviations from best Practice’ One of the subthemes derived from this study, included ‘Responding to nurse deviations from best practice’, such as second nurse medication checks for a controlled drug. Participants identified that this has the potential to compromise patient safety. Participants reported their concerns were often disregarded by senior nurses or mentors, especially when voicing concerns about deviations from policy or evidence‐based practice. Graduate nurses identified a variety of bullying behaviours That negatively impacted on teamwork and communication, thereby affecting patient safety.	CASP qualitative checklist 90%
Willman et al. (2021) Sweden ‘Insufficiently supported in handling responsibility and demands: Findings from a qualitative study of newly graduated nurses’	To explore newly graduated registered nurses' experiences and how they manage complex patient situations	Qualitative inductive	16 graduate nurses from acute care hospitals in central Sweden after a mandatory clinical development programme	Four focus groups, semi structured Latent content analysis	Major theme: ‘Not being sufficiently prepared and supported to meet responsibilities and demands’. Three sub‐themes: ‘Responsibility is not in proportion to competence’, ‘Lack of medical competence and experience complicates patient safety’ and ‘Strives for control to manage and organise nursing care’ Participants reported a lack of preparedness for managing medications safely in complex patient situations, particularly for those with a large list of medications. Participants also reported a lack of critical thinking/judging of prescriptions. Participants reported not having time to read carefully or reflect on medication, creating uncertainty and fear of doing the wrong thing. Nursing care for complex patient situations was increasingly difficult with lack of experienced staff on the ward and with having to supervise the next newly graduated nurses and/or student nurses. Participants were more concerned with medication administration but less concerned with side effects, education, interaction s or effects on patient health.	CASP Qualitative checklist 90%
Wilson et al. (2016) Australia ‘Interprofessional collaborative practice for medication safety: Nursing, pharmacy, and medical graduates' experiences and perspectives’	To report the perspectives and experiences of recently graduated Australian nurses, pharmacists and doctors in relation to interprofessional collaborative practice (IPCP) and medication safety	Qualitative interpretive research	Of the 68 health graduates, 28 were graduate nurses from three Australian states	12 focus groups semi structure Thematic analysis	Five themes: ‘Knowing what the others can do’, ‘Valuing each other and feeling valued’, ‘Respecting each other and feeling respected’, ‘Communicating with each other’ and ‘Collaborating towards a common purpose’. Participants identified that poor communication and passive aggressive behaviours resulted in medication errors or near misses. A major theme that was derived from the data included the Lack of knowledge and understanding of interprofessional abilities and responsibilities regarding medication safety. This often resulted in confusion and frustration around overlapping responsibilities, as well as missed opportunities to collaborate. Open and direct communication, as well as mutual valuing of roles, respect and trust were important in IPCP medication safety. Interprofessional education should be introduced on clinical placement for all health professional students, including the opportunity to learn about the roles and responsibilities of other professionals.	CASP Qualitative checklist 70%

Abbreviation: IPCP, Interprofessional Collaborative Practice.

**TABLE 2:** Data matrix of quantitative findings.

Author/year/country/title	Study aim	Study design	Population and setting	Measures/analysis	Main findings	Risk of bias tool
Chan and Burns (2021) USA ‘Quantifying and remediating the new graduate nurse resident academic‐practice gap using online patient simulation’	To quantify the academic practice gap and to strengthen areas of development for safe clinical practice through an online patient simulation	Quantitative quasi‐experimental pretest–posttest design Educational intervention occurred over 23 weeks	29 nurse residents (< 1 year clinical experience)	Variables specific to simulation scenario including: pretest and posttest clinical case pass rates, no. of sentinel events/causes, medicationerrors/types Descriptive statistics (frequency)	All participants (*n* = 29) caused at least one sentinel error event in the pretest phase, with a total of 766 medication errors committed. The most common medication error type consisted of antihypertensive medications and intravenous (IV) administration medications. Following the intervention, 166 medication errors occurred with an 81% reduction in IV administration errors, an 88% reduction in IV push medication errors and an 89% reduction in blood product errors. The number of sentinel events decreased from 111 (preintervention) to 8 (postintervention).	JBI Checklist for quasi‐experimental studies 67%
Fernandez et al. 2023 Turkey, South Africa, India and Australia ‘Predicting behavioural intentions towards medication safety among student and new graduate nurses across four countries’	To identify the behavioural intentions of nursing graduates' and final year nursing students' intention towards making a medication error	Quantitative multisite cross sectional study	486 new graduate nurses from Australia and Turkey (Data compared with student nurses from 4 countries)	Theory of Planned Behaviour‐Medication Safety questionnaire (TPB‐MSQ) Descriptive statistics/inferential/one way ANOVA/multiple linear regression	Study found Australian and Turkish graduate nurses to have significantly higher scores on all TPB variables (Attitude, perceived behavioural control, subjective norms and decision difficulty) compared to nursing students from all four countries (*p* = 0.00). In a comparison between Australian and Turkish new graduate nurses, Australian new graduates’ nurses reported significantly higher scores in intention to behave in a manner promoting medication safety (mean: 3.23, SD: 0.61, *p* = 0.00), better attitudes towards medication management (mean: 5.38, SD: 0.48, *p* = 0.00), greater perceived behavioural control (mean: 5.42, SD: 0.66, *p* = 0.01) and higher subjective norm scores (mean 5.52, SD 0.63, *p* = 0.00). A multiple regression model to predict all new graduate nurses' intention to practice safe‐medication management was significant (17.7% of the variance, *p* < 0.0001). Attitudes towards medication management were found to be significantly related to intention to practice safe‐medication management (*p* < 0.0001).	JBI Checklist for analytical cross sectional studies 86%
Sahay et al. (2015) Australia ‘Exploring the influence of workplace supports and relationships on safe medication practice: A pilot study of Australian graduate nurses’	To investigate the association between workplace supports and relationships and safe medication practice among graduate nurses	Quantitative descriptive study of a larger mixed methods study	58 nursing graduates in two Australian States with Most were from medical/surgical, acute or community care settings	Quantitative survey of nature of medication errors, erosion of safe medication practice and nature of medication reporting (Mixture of dichotomous, open ended, 16‐item Likert scale, multiple choice) Descriptive statistics/Spearman's correlation analysis	58.6% of the sample reported making medication errors, with most medication errors being the wrong time (29.3%), omission of medication (27.6%) and wrong dose (22.4%) 24.1% of participants did not report medication errors they had made or witnessed. A quarter of the sample disclosed they did not report witnessed medication errors as a result of being bullied by other nurses Statistically significant relationships were found between supportive Nurse Unit Manager (NUM) relationships and erosion of graduate safe‐medication practice (*r* = −0.46, *p* < 0.001); along with supportive work team behaviours and erosion of graduate safe‐medication practice (*r* = −0.51, *p* < 0.001); and education and learning support and erosions of graduate safe‐medication practice (*r* = −0.34, *p* < 0.001). Positive Spearman's correlations were found between disruptive nurse behaviour and erosion of graduate safe‐medication behaviour (*r* = 0.68, *p* < 0.001) and with disruptive physician behaviour (*r* = 0.52, *p* < 0.001). This signifies that an increase in disruptive nurse and physician behaviour or hostility impacts on medication safety among graduate nurses	JBI Checklist for analytical Cross‐sectional studies 100%

Abbreviations: IV, intravenous; NUM: Nurse Unit Manager; TPB‐MSQ; Theory of Planned Behaviour‐Medication Safety Questionnaire.

**TABLE 3:** Data matrix of mixed‐methods findings.

Author/year/country/title	Study aim	Study design	Population & setting	Measures/analysis	Main findings	Risk of bias tool
Lim and Honey (2017) New Zealand ‘New graduate nurses' knowledge and skills in medication management: Implications for clinical settings’	To explore new graduate nurses' perceptions of applying their pharmacology knowledge to clinical practice	Mixed‐methods	25 graduate nurses in New Zealand hospitals	Pharmacology knowledge tool using a 5‐point Likert scale measuring rationale, application, mechanism of action, monitoring, medication clearance, dosing, formulation, adverse effects, predictable side effects, patient education, medication information and evaluation. Descriptive statistics/thematic analysis for open‐ended questions	**Quantitative findings:** A total of 88% of respondents ‘always’ or ‘often’ felt confident in finding resources about medications, correct dosages and understood why medications were given. A total of 76% of participants reported they knew the side effects of the medications. Only 40% of participants indicated that they ‘sometimes’ or ‘seldom’ considered the clearance of the medication with 32% of participants indicated they ‘sometimes’ when explaining the mechanism of the medication. **Qualitative findings:** Two themes were identified: ‘Impediments to effective medication management’ and ‘Ways to improve medication management and safety’. Four subthemes were discussed under the first major theme and included ‘A Lack of time and a heavy workload’, ‘A lack of pharmacology knowledge’, ‘Resources’ and ‘A lack of experience’. Potential improvements for the future included improvement of pharmacology knowledge at an undergraduate level. Participants identified that having ongoing clinical education, particularly medications commonly used in their area of practice was helpful to medication management	MMAT 71%
Myroniak and Elder (2021) USA ‘Improving safe medication administration in new RNs using simulation’	To explore the use of simulation on safe‐medication administration, knowledge and skill in new registered nurses	Mixed methods, quasi‐experimental post test design	54 new RNs from a large healthcare system in the Midwest	Medication administration knowledge test specific to healthcare setting Modified Medication Administration Safety Assessment Tool by Goodstone and Goodstone (2013) (simulation performance) Debriefing session (PEARLS Healthcare Debriefing Tool by Bajaj et al., 2017).	**Quantitative findings:** Simulation slightly increased medication administration knowledge posttest scores (*M* = 11.79, SD = 2.42) compared to the control group of didactic content (*M* = 10.56, SD = 2.4), however these results were not statistically significant (*p* = 0.056). Perceived reasons for medication errors by novice RNs include interruptions, communication errors and inadequate staffing levels, with timing errors (51.9%) and medication omissions (23.2%) identified as the most common type of errors. A fear of retribution (73.3%) and not wanting to appear incompetent (62.6%) were the most common reasons for new RNs not reporting medication errors, with 47.1% of participants not realising an error had occurred. **Qualitative findings:** All groups made the same error regarding an incorrect IV push, with no groups questioning the wrong dose or using resources to look up safe dose ranges for the unfamiliar medication. Recommendations by novice registered nurse's include a desire for medication administration education earlier into their new graduate programme and a review of medication administration policies during orientation	MMAT 57%
Treiber and Jones (2018a) USA ‘After the medication error: Recent nursing graduates' reflections on adequacy of education’	To understand graduate nurses perceptions on adequacy of nursing education, their experiences after making a medication error and the organisation's response.	Mixed‐methods	168 Graduate nurses	Multipart 12‐item survey including demographics, perceptions of preparation adequacy, medication administration errors including description, contributing factors, feelings and how the nurse was treated after making the error.	Quantitative findings: Of the 168 participants 55% reported that they have made a medication error since becoming a registered nurse 24% of participants reported that they did not report the medication error. Main reasons included protecting themselves from shame and disciplinary actions. **Qualitative findings:** A major theme that was identified was the request of more practice with medication administration. Participants expressed they wanted more practice administering IV medications and more high‐risk medications. Another theme was real world versus classroom. Many participants expressed they felt unprepared to handle the stress and demands of giving multiple medications to multiple patients. One stating ‘*more focus on time management and how to handle stress*’ another stating ‘*focus more on what real nursing, that is*, *many distractions, multiple patients and phone calls*’ Multiple participants stating they wanted training in coping with making a medication error such as prepare them for doctors or pharmacists ordering errors and ordering wrong drug or dose. Others requesting to have a guest speaker talk about actual errors made and resolved so nurses could prepare for practice. Many participants stating that ‘being new’ was the major contributing factor increasing medication errors, others stating feeling overwhelmed, high patient acuity, multiple medication and low staffing were also contributing factors. Other mentioned being fatigued and higher chance of errors in the end of their shift. Rushing and hurrying due to time restraint and busy workloads contributed to errors performed. Time pressures also linking with loss of focus and distractions. One stating ‘*being too busy to follow the five rights in totality*’. Participants discussed lack of familiarity with technology, especially in the event of malfunctioning, enhances medications errors	MMAT 71%
Treiber and Jones (2018b) USA ‘Making an infusion error: The second victims of infusion therapy related medication errors’	To discuss second victim syndrome from an infusion therapy‐related medication error and its impacts on nurses	Quantitative cross‐ sectional, descriptive survey design	168 responses from graduate nurses	Quantitative survey of the incident and impacts of medication errors (Mixture of dichotomous, free‐form text and multiple choice) Descriptive statistics and key themes identified	**Quantitative findings:** 56% of respondents reported making at least one medication error since becoming an RN, with more than one third of the medication administration errors related to infusion therapy (37%). The most common IV medication errors included antibiotics, anticoagulants and antianxiety agents, with most occurring due to incorrect dosage errors. **Qualitative findings:** Analyses demonstrated that nurses frequently blamed themselves for failing to double check medications. Contributing factors included general inexperience with IV medication administration, fatigue, time pressures and involvement in high‐stress emergency situations. Overriding the medication administration system also resulted in dosage errors or multiple doses administered. An infusion error often resulted in a positive lesson learned, however, some nurses discussed negative emotional responses following a medication error	MMAT 71%

### 5.3 Data Evaluation

The quality appraisal scores for each included article are displayed in each of the data matrices, as independently evaluated by the authors. Studies that received a lower score were not excluded from this review; however, the findings from these studies were carefully considered in line with current literature. As demonstrated in Table 1, one qualitative study was considered by the authors to have a lower CASP score because of the lack of clarity around the finalisation process of the themes (Wilson et al. 2016). Similarly, the findings from studies with small sample sizes, such as that of Chan and Burns (2021), must also be interpreted with caution, in which inferences are unable to be applied to other nurse graduate populations. Most of the mixed‐methods design studies received reduced MMAT ratings because of the lack of rationale or explanations behind the use of a mixed‐methods design and/or the integration of mixed‐methods findings. Regardless of these limitations, the authors consider the information provided in the data matrices to be representative of the literature addressing the research questions.

### 5.4 Frequency and Common Types of Medication Errors

The increased magnitude of observed or self‐reported MAEs among novice registered nurses was demonstrated in this review. Multiple studies reported that over half of their participants made an MAE in their first year of nursing (Myroniak and Elder 2021; Sahay et al. 2015; Treiber and Jones 2018b). Interestingly, in two studies, nearly one‐quarter of participants did not report MAEs that they had made or witnessed, which suggests that the true number of errors could be higher (Sahay et al. 2015; Treiber and Jones 2018a). One study reported a total of 766 medication errors in the preintervention phase of a simulated scenario by novice registered nurses (Chan and Burns 2021). Following the educational intervention, a total of 166 medication errors and eight sentinel events were reported, indicating that the education provided was effective with room for further improvement (Chan and Burns 2021).

The common types of MAEs among novice registered nurses identified by researchers, ranked in order from highest to lowest, were wrong time, missed medication instances, wrong dose, incorrect route, administration of incorrect medications, errors in patient identification and lapses in error recognition (Chan and Burns 2021; Lim and Honey 2017; Myroniak and Elder 2021; Sahay et al. 2015; Treiber and Jones 2018a, 2018b). Four studies highlighted frequent errors with intravenous (IV) administration and high‐risk medications such as antihypertensives, antibiotics, anticoagulants and antianxiety agents (Chan and Burns 2021; Myroniak and Elder 2021; Treiber and Jones 2018b). Notably, Treiber and Jones (2018b) revealed that 56% of 168 novice registered nurses reported MAEs, with over a third of these errors involving IV medication administration. Interestingly, a simulation study reported that all groups made the same error when administering an IV push medication, delivering an incorrect dose outside safe ranges (Myroniak and Elder 2021). All participants questioned the diluting volume; however, none questioned the dosage or used resources to look up the safe dose range for morphine (Myroniak and Elder 2021).

### 5.5 Influencing Factors of Medication Errors

#### 5.5.1 Intrinsic Factors

The major intrinsic factors contributing to MAEs by newly registered nurses were identified as lack of confidence, poor coping skills and reluctance to approach experienced colleagues. As expressed by one graduate participant ‘being new with lots of pressure to be perfect’ (Treiber and Jones 2018b) contributed to participants' loss of confidence, ultimately affecting medication administration and safety. Additionally, most studies reported that insufficient exposure to medication administration during undergraduate nursing programmes exacerbated nervousness and created barriers to effective medication administration (Fernandez et al. 2023; Lim and Honey 2017; Sahay et al. 2015; Treiber and Jones 2018a). The lack of practical experience with medications during undergraduate training was consistently associated with heightened anxiety and a greater risk of MAEs among novice nurses. Multiple researchers highlighted that exposure to high‐risk medications is needed in the undergraduate Bachelor of Nursing programme to improve the confidence of novice registered nurses when administering these medications (Fernandez et al. 2023; Lim and Honey 2017).

#### 5.5.2 Extrinsic Factors

Extrinsic factors were the most frequently reported cause of MAEs by novice registered nurses in this review. Five studies highlighted time pressures, hectic workloads, low staffing, and high‐stress environments as significant contributors (Lim and Honey 2017; Sahay and Willis 2022; Treiber and Jones 2018a, 2018b; Willman et al. 2021) with Treiber and Jones (2018a) also identifying that polypharmacy for high acuity patients was a major factor. One participant reflected ‘the challenges in the real world of nursing practice go beyond simple knowledge of medications…having several patients, other nurses in the medication room… family members and physicians with comments and questions… very overwhelming… very different than passing an examination about dosage calculations’ (Treiber and Jones 2018a). This indicates the need for an authentic learning experience for medication administration. Additionally, participants felt they had a reduced capacity to guarantee patient safety because of time pressures and heavy workloads. One participant mentioned being ‘too busy to stop and go to look up information’ regarding an unfamiliar medication, ultimately resulting in a medication error (Lim and Honey 2017).

Another sub‐theme highlighted in the studies is the impact that bullying and work culture can have on the frequency of MAEs among novice registered nurses. Intimidation, pressure, condescending language and harsh tones from senior nursing staff and medical practitioners, were often identified behaviours leading to increased stress and enhanced emotional strain impacting medication administration (Myroniak and Elder 2021; Sahay et al. 2015; Sahay and Willis 2022; Treiber and Jones 2018a). This was highlighted in Treiber and Jones (2018a) where a participant stated that they ‘felt pressure by a coworker… new on a busy unit… the nurse I was helping was very intimidating… fear of making a bad impression clouded my judgement’. Similarly, one participant stated, ‘*horizontal violence impairs our clinical decision making*’ (Sahay and Willis 2022), which indicates the importance of a supportive work environment.

Culture and bad habits within organisations were also discussed in multiple studies, where deviations from best practice were the most prominent factor in contributing to medication (Sahay et al. 2015; Sahay and Willis 2022; Treiber and Jones 2018a). Most newly registered nurses observed deviations from best practice by senior staff; however, due to inexperience and lack of confidence, they did not speak up (Sahay et al. 2015; Sahay and Willis 2022). A common discrepancy from best practice involved omitting the required second RN checks for medications, particularly when accompanying nurses to the bedside to complete the medication check (Sahay and Willis 2022). Novice registered nurses need to be encouraged to adhere to best practices in medication safety, especially in the event of incivility.

### 5.6 Clinical, Educational and/or Research Implications

#### 5.6.1 Simulation and Authentic Learning Experiences

Multiple studies have investigated the correlation between the lack of simulation in Bachelor of Nursing programmes and MAEs committed by novice registered nurses (Chan and Burns 2021; Lim and Honey 2017; Myroniak and Elder 2021; Treiber and Jones 2018a). Several novice registered nurses reported feeling unprepared for the stress and demands of managing multiple patients and administering numerous medications, highlighting the need for enhanced education and simulation to mitigate the risk of errors (Treiber and Jones 2018a). Participants emphasised the necessity of focusing on the realities of nursing, suggesting that incorporating simulated distractions, multiple patients and phone calls could help reduce the incidence of errors (Treiber and Jones 2018a). Studies that utilised pre‐ and posttesting to evaluate the impact of simulation reported significant improvements following the implementation of simulation training (Chan and Burns 2021; Myroniak and Elder 2021). One study even reported a 73% reduction in MAEs after the introduction of simulation programs (Chan and Burns 2021). The education provided during simulated scenarios or during debriefing should include how MAEs occur, the consequences of these errors, and strategies to avoid these errors (Fernandez et al. 2023; Myroniak and Elder 2021; Treiber and Jones 2018b).

#### 5.6.2 Deficiency in Pharmacology Knowledge

A predominant theme throughout the papers in this review was the lack of pharmacology knowledge. Medication administration errors were often linked to unfamiliarity with high‐alert medications, limited knowledge about the reasonableness of prescriptions and inadequate understanding of the mechanisms of medications (Lim and Honey 2017). In two studies, participants stated that more pharmacological education should be provided early in undergraduate nursing programmes and continue through the transition into nursing practice. One participant suggested that undergraduate education institutes should increase pharmacology content in their curriculums to better prepare students for the role as a registered nurse (Lim and Honey 2017; Myroniak and Elder 2021). The most frequently identified area for improvement was the delivery of pharmacological education in both undergraduate programmes and ongoing in their work environments. Participants emphasised the need for specific education on drug mechanisms of action, drug clearance and pharmacological principles (Lim and Honey 2017; Treiber and Jones 2018a; Willman et al. 2021). Given that IV medications delivery resulted in the highest number of MAEs (Treiber and Jones 2018b), ongoing education on IV medication administration is crucial for patient safety.

#### 5.6.3 Future Research Implications

Several areas within the studies were highlighted as requiring further investigation to understand and reduce MAEs among graduate nurses. Multiple studies emphasised the importance of incorporating simulation into undergraduate nursing education and reported that authentic simulations, such as handling multiple patients and medications, reduced MAEs (Chan and Burns 2021; Fernandez et al. 2023; Kass et al. 2018; Myroniak and Elder 2021; Treiber and Jones 2018a). Given that some of these studies involved smaller cohorts, further investigation into simulation medication administration scenarios with larger student groups is recommended.

There is a recognised need for targeted education and support to help identify and manage medication errors within undergraduate programmes. A quantitative study indicated promising benefits from debriefing, access to learning packages, education sessions and pairing students with experienced nurses. However, further research is necessary to evaluate the impact on newly registered nurses (Sahay and Willis 2022). Interestingly, the timing of education delivery within the undergraduate nursing curricula may be a critical factor. Lim and Honey (2017) noted that many curricula's delay pharmacology education until later in the nursing programme. Future research should explore the benefits of introducing pharmacology education in the first year of undergraduate nursing studies and progressively build on this pharmacology knowledge throughout the entire programme.

## 6 Discussion

Although a wealth of literature exists on medication administration errors (MAEs) in hospital settings, there is limited evidence specific to newly registered nurses. This is significant because evidence indicates that newly registered nurses are responsible for a higher number of MAEs compared to their more experienced counterparts (Björkstén et al. 2016; Keers et al. 2018). This integrative review specifically examined research on the factors contributing to MAEs made by novice registered nurses, an important area given the WHO's strategic framework of the *Global Patient Safety Challenge*, which identifies education and training of new healthcare workers as essential to medication safety (World Health Organization 2023). Identifying these factors allows for recommendations to improve training and medication administration practices. Contributing factors to MAEs by newly registered nurses included intrinsic and extrinsic factors, the need for both simulated and authentic learning experiences and a lack of pharmacological knowledge. The review also highlighted areas requiring further investigation.

### 6.1 Importance of Pharmacology Education

A lack of pharmacological knowledge and confidence in medication calculations is perceived to contribute significantly to medication administration errors, as indicated by the studies reviewed. Research has shown that nurses' confidence, experience levels and prior education are critical factors that can lead to administration errors, monitoring errors and medication interactions (Escrivá Gracia et al. 2019; Lim and Honey 2017; Wondmieneh et al. 2020). For instance, Lim and Honey (2017) underscored the importance of extending pharmacology education into the graduate healthcare setting, suggesting the incorporation of annual updates for registered nurses. This view is echoed by Di Simone et al. (2018), who emphasised the necessity for strong calculus skills and highlighted that younger, inexperienced nurses often lack the confidence and skills in medication calculations compared to their more experienced colleagues.

Furthermore, Lim and Honey (2017) targeted education programmes designed to address the unique medication‐related needs of each hospital ward setting. This approach involves collaboration among various healthcare professionals, including pharmacists and medical officers, to deliver tailored educational interventions focused on the most frequently used and high‐risk medications within each area. Customising medication education could enhance medication safety protocols and optimise patient outcomes. Similar responses are reflected in other studies, such as those by Di Simone et al. (2016) and Di Simone et al. (2018) which support the need for such department‐specific education.

Interestingly, Lim and Honey (2017) and Myroniak and Elder (2021) proposed that pharmacology education should begin early in undergraduate nursing programmes to enhance knowledge retention. They suggested that implementing basic pharmacology content in the first year and gradually progressing to more complex and high‐risk medications by the final year could potentially close the pharmacological knowledge gap. This educational model is further supported by Treiber and Jones (2018a), who found that newly registered nurses felt confident in their medication knowledge. However, they observed that this knowledge often diminished when these nurses faced the complexities of the clinical environment. Amiri et al. (2018) also emphasised the role of continuous professional development, finding that ongoing training programmes significantly reduce the incidence of MAEs.

### 6.2 Simulation and Authentic Learning Experience

Simulation and authentic learning were a common theme in this integrative review, in which multiple studies implemented a variety of simulation scenarios in both undergraduate and hospital settings. Chan and Burns (2021) integrated complex patient scenarios that replicated real‐life settings, which were reported to have substantial improvements in both medication administration errors and the confidence levels of the newly graduated nurses. Treiber and Jones (2018a) suggested that simulation should include the setting, patient charting, sounds and equipment similar to a clinical setting. Seaton et al. (2019) further discussed that simulations could be improved by incorporating more complex patient scenarios, such as multiple medication orders, and including authentic distractions from a clinical setting, such as call bells, alarms and realistic patient ratios. Interestingly, Treiber and Jones (2018b) discussed how several participants reflected on their MAEs in a simulated setting and noted that such experiences were invaluable learning opportunities. Consequently, they demonstrated a marked improvement in avoiding the same errors in real clinical scenarios, having already confronted and understood the implications of their actions without the added concern of causing harm to patients. Furthermore, simulation and training instilled greater confidence in newly registered nurses regarding error reporting and subsequent action‐taking, as they had already encountered such scenarios in simulated environments. Both simulation and pharmacological education were identified as influencing factors in the undergraduate programme to improve MAEs; however, more research is required about its implementation in larger cohorts.

### 6.3 Technologies and Transition

Notably, the role of technology and the use of electronic medical records (EMRs) was largely missing from this review. The readiness of novice registered nurses to use EMRs has been previously highlighted as a significant concern, with evidence suggesting newly registered nurses are underprepared for EMR use (Mollart et al. 2020). This is concerning given the widespread implementation of EMRs in the clinical environment. Current research has found that EMRs contribute to reducing medication errors and improving communication among healthcare professionals (Mollart et al. 2020, 2021). Therefore, further research is warranted on incorporating EMR training into undergraduate programmes and its impact on medication administration safety for newly registered nurses. Mollart et al. (2021) identified that a lack of confidence and competence in using EMR is a significant issue for novice registered nurses, emphasising the importance of integrating EMR education into undergraduate curricula to enhance medication safety. Raghunathan et al. (2021) also identify the need to examine the impact of EMR use on critical thinking and clinical performance, particularly in novice registered nurses.

The transition from undergraduate to registered nurse can be complex and intensive, necessitating competence in key clinical areas such as medication administration (Mollart et al. 2021). Although mathematical competency is an important element of medication administration, a scoping review by McKenna et al. (2022) identified that nursing students experienced increased anxiety during medication calculations. This is significant, as incorrect dosages were identified as a common type of MAEs from this review. Currently, there is minimal literature linking the mathematical competency of undergraduate nursing students and errors in calculating medication dosages (McKenna et al. 2022). Therefore, future research could provide valuable insights for enhancing the undergraduate nursing curricula in this area.

### 6.4 Enhancing Nontechnical Skills in Novice Registered Nurses

Numerous studies have demonstrated that improving nontechnical skills, such as communication and situational awareness, can enhance patient safety during medication administration by newly registered nurses (Wilson et al. 2016). Key areas identified for further research include safety‐oriented communication, leadership, mentorship, situational awareness and understanding interprofessional dynamics, particularly in pharmacology. Despite the substantial literature on the subject, mentoring and preceptorship were notably absent from this review. Preceptorship aims to welcome, orientate and support novice registered nurses in the clinical environment (Lafrance 2018). Preceptorship relationships between experienced nurses and newly registered nurses have been shown to enhance skills and practical knowledge in areas such as medication administration (Lafrance 2018). However, this concept was not identified as a mitigating factor in this review. Given the existing literature promoting the benefits of leadership and preceptors on newly registered nurses, the role of mentorship in reducing MAEs warrants further investigation.

## 7 Strengths and Limitations

A notable strength of this review is the focus on novice registered nurses. To the best of the authors' knowledge, no prior review has been conducted on this topic. The integrative review methodology is another strength of this study because it synthesises both quantitative and qualitative research, providing a comprehensive understanding of medication administration errors. This approach allows for the inclusion of a wider range of studies, thereby enhancing the reliability and applicability of the findings. Medication errors represent a persistent issue in healthcare, prompting extensive research over the years. However, this review exclusively considers studies published after 2013, potentially omitting pertinent but older data. Additionally, limiting the review to English language studies may have further narrowed the scope of analysis.

It is important to note that this review specifically targeted newly graduated novice nurses, thereby excluding literature on experienced registered nurses, enrolled nurses, and other healthcare professionals involved in medication administration. Given that the focus was solely on medication administration errors, key search terms were utilised to this effect, which may have inadvertently resulted in the omission of studies that addressed broader transitional issues affecting novice registered nurses.

## 8 Conclusion

This review highlighted several factors influencing MAEs among newly graduated nurses. Both intrinsic and extrinsic factors, such as individual confidence and workplace environment, play significant roles. Additionally, the lack of simulation training and authentic learning experiences, coupled with deficiencies in pharmacological knowledge, was found to contribute substantially to MAEs. The findings suggest that improving education, fostering a positive ward culture and providing strong support for graduates can significantly reduce errors. Implications for clinical practice, education and future research were also identified. Moreover, EMR's impact on medication administration is a critical area for future research. With EMR widely adopted in health care, studying its effects on newly graduated nurses could enhance patient safety and outcomes. Addressing this gap could help to mitigate MAEs and improve care quality by novice registered nurses.

## Author Contributions

A.M. and K.M. conceptualised the review with C.W. and E.D. selecting the methodology. C.W. and E.D. completed data collection, quality appraisal and data extraction with K.M. aiding in the event of any disagreements. All four authors contributed to drafting and editing the manuscript. All four authors have read and agreed to the published version of the final manuscript.

## Conflicts of Interest

The authors declare no conflicts of interest.

## Data Availability

The data that support the findings of this study are available from the corresponding author upon reasonable request.
